# Carcass Traits, Meat Quality, Hydrolyzed Amino Acid Composition, and GC–IMS Volatile Signal Profiles in Kazakh Lambs Fed Diets Containing Fermented Cottonseed Hulls

**DOI:** 10.3390/foods15203739

**Published:** 2026-10-20

**Authors:** Caidie Wang, Honghai Yang, Xiaoyan Zhou, Hanzuohere Yishake, Songcheng Li, Yan Liu, Hongman Li

**Affiliations:** Xinjiang Herbivore Nutrition Laboratory for Meat & Milk, College of Animal Sciences, Xinjiang Agricultural University, Urumqi 830052, China; 320242693@stu.xjau.edu.cn (H.Y.); nice2cool@163.com (X.Z.); 18099337170@163.com (H.Y.); 17590060365@163.com (S.L.); l2077845189@163.com (Y.L.); 320252793@stu.xjau.edu.cn (H.L.)

**Keywords:** fermented cottonseed hulls, Kazakh lambs, meat quality, hydrolyzed amino acids, candidate volatile signals, GC–IMS

## Abstract

Cottonseed hulls, a fibrous by-product of cotton processing, are a potentially valuable unconventional feed resource for ruminants, and microbial fermentation may improve their feeding value. This study preliminarily compared plasma antioxidant status, slaughter performance, meat quality, hydrolyzed amino acids, and tentatively assigned GC–IMS volatile signals among Kazakh lambs fed diets containing wheat straw (WS), cottonseed hulls (CSH), or fermented cottonseed hulls (FCSH). Thirty male lambs were allocated to three groups (10 per group), with one pen per diet; six lambs per group were selected for slaughter. At the animal level, plasma biochemical indices showed no nominal between-group differences, whereas T-SOD activity was nominally higher in CSH and FCSH than in WS. Among slaughter-selected lambs, the FCSH group had nominally higher pre-slaughter live weight and carcass weight than the WS and CSH groups, with no detectable differences in dressing percentage or conventional meat-quality traits. Muscle crude fat was nominally higher in the FCSH group than in the WS group, but not different from the CSH group. TAA, EAA, and EAA/TAA showed no nominal between-group differences. Four individual hydrolyzed amino acids and 11 of 66 GC–IMS signals had unadjusted *p* < 0.05, but none reached *q* < 0.05 after Benjamini–Hochberg FDR correction. Because each diet was represented by only one pen, treatment and pen effects cannot be separated, and these animal-level findings should be considered exploratory and require validation with independent pen replication.

## 1. Introduction

Sheep meat is the principal end product of the meat-sheep industry, and its production efficiency, nutritional value, and eating quality jointly determine farming profitability and market competitiveness. The characteristic odor and flavor of sheep meat are important factors affecting consumer acceptance, while feeding system, diet composition, breed, and postmortem processing conditions can all influence these quality attributes; diet, in particular, can regulate fatty acid composition, antioxidant constituents, and volatile substances [1]. Therefore, improving feed-resource utilization through dietary strategies while maintaining animal performance and meat quality is an important goal in meat-sheep nutrition research and production. This is particularly relevant in regions rich in unconventional feed resources, where converting agricultural processing by-products from low-value fibrous materials into roughage resources with higher feeding value can reduce dependence on high-quality roughages and improve regional feed-resource utilization. Cotton (*Gossypium* spp.) processing generates large quantities of by-products, including cottonseed hulls, cottonseed meal, and cotton stalks. Among these, cottonseed hulls (CSH) are characterized by a stable supply and high fiber content and have potential value in ruminant diets. However, their nutrient structure also limits further utilization. Bo et al. [2] reported neutral detergent fiber and acid detergent fiber concentrations of 716.4 and 464.1 g/kg DM, respectively, in linted CSH, whereas crude protein content was relatively low. Zanine et al. [3] observed declines in dry matter intake, digestibility of some nutrients, and eating and rumination efficiency as the level of untreated cottonseed hulls increased in ram diets. CSH may also contain residual free gossypol, and high intake may adversely affect animal growth, organ function, and reproductive health [4].

Microbial solid-state fermentation can improve the feeding quality of cotton by-products by degrading fibrous structures, reducing antinutritional factors, and accumulating organic acids, enzymes, and low-molecular-weight metabolites. A study screening different fungi for solid-state fermentation of cottonseed meal showed that fermentation with *C. capsuligena* ZD-1, *C. tropicalis* ZD-3, *S. cerevisiae* ZD-5, *A. terricola* ZD-6, *A. oryzae* ZD-7, or *A. niger* ZD-8 reduced free gossypol and improved nutritional value [5]. Zhang et al. [6] subsequently optimized a microbial fermentation detoxification process and improved the in vitro utilization value of protein and amino acids in cottonseed meal. Yin et al. [7] screened the gossypol-degrading fungus *Aspergillus oryzae* TM-2; during solid-state fermentation of CSH, the gossypol degradation rate reached 69.54%, crude protein and acid-soluble protein increased, and cellulose, hemicellulose, lignin, NDF, and ADF decreased. Inclusion of fermented cotton by-products in animal diets may further affect growth performance and muscle quality. Existing studies show that solid-state fermentation can effectively improve the feeding quality of CSH. Du et al. [8] found that solid-state fermentation of CSH markedly reduced free gossypol and condensed tannins, improved nutrient composition, and promoted degradation of lignocellulosic structures. Existing animal studies provide preliminary support for using fermented cotton by-products in ruminant diets. Ren et al. [9] reported that replacing corn silage with fermented cottonseed hulls (FCSH) improved some growth-performance indices in finishing Kazakh sheep and altered plasma urea nitrogen, AST, and ALT, whereas slaughter traits did not differ significantly. Yusuf et al. [10] reported that a fermented total mixed ration containing fermented cottonseed meal maintained growth performance in Hu sheep and altered some rumen fermentation and meat-quality-related indices. Wei et al. [11] found that cotton stalks fermented with compound probiotics improved nutrient utilization and rumen microbiota in sheep. Recent studies further indicate that fermented diets or microbial interventions can affect not only rumen microbiota and fermentation characteristics in lambs but also muscle fatty acid composition and volatile-compound profiles. Liu et al. [12] found that different forage proportions in fermented total mixed rations altered muscle fatty acid composition and rumen microbiota in lambs; Li et al. [13] reported that yeast-culture intervention simultaneously affected rumen fermentation, intramuscular fat, and volatile-compound composition; and Wang et al. [14] further found that flavor precursors such as fat, fatty acids, and free amino acids were closely associated with volatile compounds in cooked lamb. Muscle lipids and their fatty acid composition are important precursors of volatile aroma formation in lamb. Li et al. [15] showed by lipidomic and volatilomic analyses that differences in intramuscular fat content and lipid composition in Hu sheep were accompanied by changes in volatile aroma profiles, some of which may be related to thermal oxidation of unsaturated fatty acids. Chang et al. [16] recently further noted that interactions between gastrointestinal microbiota and lipid metabolism may participate in the formation of lamb flavor precursors such as fatty acids and amino acids. Regarding specific microbial strains, Wang et al. [17] supplemented Sunit sheep diets with a compound lactic-acid-bacterial preparation containing *Lactiplantibacillus plantarum* and *Lacticaseibacillus casei*; the lower supplementation level reduced shear force and cooking loss, whereas responses at the higher level were inconsistent. Thus, besides potentially improving roughage utilization, fermented cottonseed hulls may affect muscle quality by altering nutrient supply and fermentation metabolites. In our previous work, CSH were subjected to compound solid-state fermentation with *Aspergillus niger*, *Aspergillus oryzae*, and *Lactiplantibacillus plantarum*. Compared with untreated CSH, fermentation decreased pH and NH3-N/total nitrogen, increased lactic acid, further decreased NDF and ADF contents, and significantly reduced free gossypol; untargeted metabolomics also revealed the production of several bioactive substances [18]. However, existing studies have focused mainly on detoxification and nutritional evaluation of cottonseed by-products, and systematic research is still lacking on how replacing conventional roughage with FCSH affects muscle quality, nutrient composition, and volatile-compound formation.

Accordingly, this study used male Kazakh lambs to preliminarily compare three dietary roughage treatments with respect to plasma biochemical and antioxidant status, slaughter performance, organ indices, conventional meat quality, the hydrolyzed amino acid composition of the longissimus thoracis, and GC–IMS volatile profiles. The study was designed to explore the potential of fermented cottonseed hulls as a roughage substitute and to provide a basis for further investigation of possible links among FCSH, muscle nutrient composition, and volatile-profile variation.

## 2. Materials and Methods

### 2.1. Preparation of FCSH

The CSH used in this study were purchased from Xinjiang Tianshan Premixed Feed Co., Ltd. (Urumqi, China). FCSH was prepared using the mixed-culture solid-state fermentation process previously optimized by our research group [18]. The fermentation substrate consisted of CSH and wheat bran at a dry-matter mass ratio of 80:20. The microbial inoculants were *Aspergillus niger* CICC 2377, *Aspergillus oryzae* CICC 2339, and *Lactiplantibacillus plantarum* LP-66. The two *Aspergillus* preparations were purchased from Shandong Hezhong Kangyuan Biotechnology Co., Ltd. (Zibo, China), and the *L. plantarum* preparation was purchased from Weifang Yihao Biotechnology Co., Ltd. (Weifang, China). According to supplier information, the spore counts of the two *Aspergillus* preparations and the viable count of the *L. plantarum* preparation were each approximately 1 × 10^10^ CFU/g. The three inoculants were mixed at a 1:1:1 ratio by mass, and the mixed inoculum was added at 0.10% of the air-dry mass of the substrate. CSH and wheat bran were thoroughly mixed, the substrate moisture content was adjusted to 55%, and the mixed inoculum was incorporated evenly. Solid-state fermentation was conducted for 15 d at 20–32 °C. The FCSH used in the animal feeding trial was prepared as a single production batch. Free gossypol was measured in both the unfermented and fermented CSH–wheat-bran mixtures according to Fan et al. [19], yielding concentrations of 280.63 and 70.88 mg/kg, respectively. The present study focused on the plasma, carcass, and muscle measurements obtained under this feeding condition.

### 2.2. Experimental Animals, Diets, and Management

The experiment was conducted from September to December 2025 at Xinjiang Huikang Animal Husbandry Biotechnology Co., Ltd. (Changji, China) and was approved by the Animal Welfare and Ethics Committee of Xinjiang Agricultural University (approval No. 2025037). No formal a priori sample-size calculation or statistical power analysis was performed before the experiment; the number of animals was determined primarily by animal availability, housing conditions, experimental-design requirements, and the study objectives. Thirty healthy 3- to 4-month-old male Kazakh lambs with similar body condition were selected, with a mean pre-adaptation body weight of 27.53 ± 1.06 kg. Animals were stratified by pre-adaptation body weight and randomly allocated within strata to the wheat straw group (WS; 27.83 ± 1.10 kg), unfermented cottonseed hull group (CSH; 27.40 ± 1.10 kg), or fermented cottonseed hull group (FCSH; 27.36 ± 1.04 kg), with 10 lambs per group. The three dietary groups were housed in three separate pens, with one pen assigned to each diet, and all lambs were individually identified. The adaptation period lasted 14 d, followed by a 60-d formal experimental period. After body-weight recording and blood collection on day 60, lambs selected for slaughter continued to receive their assigned diets for an additional 4 d and were slaughtered on day 64. The three pens were located in the same north–south-oriented barn, in adjacent central positions on the same side of the barn, approximately 1 m apart. Each pen had an indoor area of 40 m^2^, a 50 m^2^ exercise yard, and four 2.5-m feed troughs (10 m total trough length). Pens were managed using the same temperature-control and hygiene procedures and were uniformly cleaned and disinfected before the trial; vaccination and deworming followed the farm’s established procedures. Throughout the trial, lambs had ad libitum access to feed and water, and the diets were offered separately to avoid cross-contact. Experimental diets were formulated according to the Nutrient Requirements of Meat Sheep (NY/T 816-2021) [20], and their composition and nutrient levels are presented in Table 1. All diets contained 15% alfalfa and 25% of the corresponding replacement mixture on a dry-matter basis (the latter was a mixture, not pure cottonseed hulls): wheat straw–wheat bran (77:23) for WS, unfermented cottonseed hull–wheat bran (80:20) for CSH, and fermented cottonseed hull–wheat bran (80:20) for FCSH. All diets were prepared as total mixed rations and offered at approximately 10:00 and 19:00 Beijing time each day; differences in feeding time among pens did not exceed 5 min. Because the dietary regimens were visibly different, personnel involved in animal management could not be fully blinded to treatment allocation. However, coded sample management was used during post-slaughter collection, labeling, and laboratory analyses so that laboratory analysts could not directly identify dietary group allocation, thereby reducing potential observer bias.

### 2.3. Blood Sampling and Plasma Biochemical Analyses

On day 60 of the experimental period, jugular blood was collected before morning feeding into sodium-heparin tubes. Plasma was separated by centrifugation at 3500 rpm for 10 min at 4 °C and stored at −20 °C until analysis. Plasma urea, glucose (GLU), total protein (TP), albumin (ALB), triglycerides (TG), total cholesterol (TC), alanine aminotransferase (ALT), and aspartate aminotransferase (AST) were measured with a Beckman AU5821 automated biochemical analyzer (Beckman Coulter, Brea, CA, USA).

### 2.4. Plasma Antioxidant Indices

Plasma antioxidant indices, including total superoxide dismutase (T-SOD), glutathione peroxidase (GSH-Px), catalase (CAT), malondialdehyde (MDA), and total antioxidant capacity (T-AOC), were measured using commercial colorimetric assay kits from Nanjing Jiancheng Bioengineering Institute (Nanjing, China). T-SOD, GSH-Px, and CAT activities are expressed as U/mL, MDA content as nmol/mL, and T-AOC as mmol Trolox equivalents/L. Detailed assay procedures, including kit catalog numbers, reaction conditions, and analytical methods, are provided in Supplementary Methods S1.

### 2.5. Slaughter Performance and Organ Indices

Before morning feeding on day 60 of the formal experimental period, all 10 lambs in each pen were weighed. According to the prespecified slaughter-selection procedure, the lamb with the highest and the lamb with the lowest day-60 body weight in each group were excluded, after which six lambs were randomly selected from the remaining eight animals for slaughter. This procedure was intended to select slaughter animals near the central body-weight range of each group; however, excluding both extremes may have reduced observed variability, and the selected subset cannot be assumed to represent all ten lambs. The selected lambs continued to receive their assigned diets for 4 d and were slaughtered on day 64. Slaughter performance was measured according to the Technical Specification for Measurement of Production Performance of Meat Sheep (T/CAAA 080-2022) [21]. Before slaughter, lambs were fasted for 18 h and deprived of water for 3 h, and pre-slaughter live weight was recorded. Animals were stunned and exsanguinated, after which post-exsanguination body weight was recorded. After removal of the skin, head, feet, and viscera, the kidneys and perirenal fat were retained according to the predefined carcass-measurement procedure; carcass weight and the weights of the heart, liver, lungs, spleen, and kidneys were recorded. Loin-eye measurements were obtained at the 12th–13th rib region. Following Wang et al. [22], the cross-sectional outline of the loin eye was traced on tracing paper to delineate the muscle boundary and identify the maximum height and width; loin-eye area was then estimated using the formula below. Backfat thickness was measured approximately 1 cm lateral to the dorsal midline at the posterior edge of the 12th rib. The GR value was measured between the 12th and 13th ribs, 11 cm from the dorsal midline. Dressing percentage was calculated using Equation (1), loin-eye area was calculated using Equation (2), and organ index was calculated using Equation (3).

Dressing percentage (%) = carcass weight/pre-slaughter live weight × 100,(1)

Loin-eye area (cm^2^) = loin-eye height × loin-eye width × 0.7, (2)

Organ index (g/kg) = organ weight/pre-slaughter live weight × 1000,(3)

### 2.6. Meat Quality and Proximate Composition

Longissimus thoracis samples from the 9th to 12th rib region were collected after slaughter. Samples designated for 45-min postmortem measurements were analyzed within 45–60 min after slaughter; samples for 24-h measurements were stored at 4 °C. Samples for proximate-composition analysis were aliquoted and stored at −20 °C. Samples for hydrolyzed-amino-acid and volatile-compound analyses were separately aliquoted, rapidly frozen in liquid nitrogen, and stored at −80 °C for approximately 3 months. Freezing and thawing procedures were identical across the three groups.

Muscle pH, color, and shear force were measured according to the Technical Specification for Measurement of Production Performance of Meat Sheep (T/CAAA 080-2022) [21]. Muscle pH was measured at 45 min and 24 h postmortem using an H1981036 meat pH meter (Hanna Instruments Inc., Woonsocket, RI, USA); each biological sample was measured three times and the mean value was used. L*, a*, and b* were measured at the same time points using a CR-10 Plus colorimeter (Konica Minolta, Osaka, Japan), with three measurements per sample averaged. Cooking loss was determined with reference to Honikel [23] and related lamb meat-quality research [24]. Approximately 10 g of longissimus thoracis was weighed to obtain the pre-cooking mass (*W*_1_), sealed in a food-grade polyethylene bag, and heated in an 85 °C water bath for approximately 45 min. After heating, samples were cooled at room temperature for 30 min, surface moisture was removed, and the post-cooking mass (*W*_2_) was recorded. Duplicate determinations were performed for each sample. Cooking loss was calculated using Equation (4). Shear force was measured using a C-LM3B digital meat-tenderness meter (Beijing Tianxiang Feiyu Technology Co., Ltd., Beijing, China). Meat samples were cut parallel to the muscle fibers into columns with an approximately 3 cm × 3 cm cross-section and placed in resealable bags (15 cm × 10 cm), and sealed after air was expelled. Samples were heated in an 80 °C water bath until the core temperature reached 70 °C, removed, cooled to room temperature in a cool, dry place, and then sheared perpendicular to the muscle fibers. Six replicate measurements were obtained per sample and averaged. Drip loss was determined using a published hanging method for lamb [25]. Visible surface fat and connective tissue were removed from longissimus thoracis samples, which were trimmed parallel to the muscle fibers into strips approximately 5 cm × 3 cm × 2 cm and weighed to obtain the initial mass (*W*_3_). Each strip was suspended inside a container using an S-shaped hook so that it did not contact the container wall or bottom. The container was then sealed inside an air-filled food-grade or resealable bag and stored at 4 °C for 24 h. After storage, samples were removed, surface moisture was gently blotted with filter paper, and the final mass (*W*_4_) was recorded. Duplicate determinations were performed for each sample. Drip loss was calculated using Equation (5).

Cooking loss (%) = (*W*_1_ − *W*_2_)/*W*_1_ × 100, (4)

Drip loss (%) = (*W*_3_ − *W*_4_)/*W*_3_ × 100, (5)

Muscle moisture, protein, and fat were determined according to Chinese National Food Safety Standards GB 5009.3–2016 [26], GB 5009.5–2025 [27], and GB 5009.6–2025 [28], respectively. Before analysis, visible fat and connective tissue were removed from the muscle samples, which were then finely minced and homogenized. Moisture was determined by direct drying: approximately 10 g of homogenized sample was dried at 101–105 °C to constant weight, and moisture content was calculated from the mass difference before and after drying. Protein was determined by the Kjeldahl method: samples were digested at 420 °C in a sulfuric-acid/catalyst system, after which protein content was measured using a Hanon K1100 automatic Kjeldahl analyzer (Hanon Advanced Technology Group Co., Ltd., Jinan, China). For fat determination, meat samples were hydrolyzed in gently boiling 2 mol/L HCl for 1 h to release bound lipids; the hydrolysis residue was washed to neutrality and dried, and then subjected to Soxhlet extraction with petroleum ether. After solvent removal and drying to constant weight, fat content was calculated from the mass of extracted lipid.

### 2.7. Hydrolyzed Amino Acid Analysis

Hydrolyzed amino acids in the longissimus thoracis were determined according to the Chinese National Food Safety Standard GB 5009.124–2016 [29] using a SCION Artemis 6000 automatic amino acid analyzer (Techcomp Instrument Ltd., Shanghai, China). Samples were thawed in an ice-water bath at 0 °C, visible adipose tissue was removed, and the tissue was homogenized on ice. A 0.10 g portion was transferred to a 20 mL anaerobic hydrolysis tube and mixed with 10 mL of 6 mol/L HCl containing 1 g/L phenol. Tubes were frozen in liquid nitrogen for 5 min and flushed with high-purity nitrogen three times, sealed under nitrogen, and hydrolyzed at 110 °C for 22 h. After cooling, mixing, and filtration, 0.5 mL filtrate was evaporated to dryness under nitrogen at 50 °C. The residue was reconstituted in 5 mL sample diluent, filtered through a 0.22 μm membrane, and analyzed with a 50 μL injection volume.

### 2.8. GC–IMS Analysis of Volatile Signals

Volatile-signal profiles were analyzed using a FlavourSpec^®^ gas chromatography–ion mobility spectrometry (GC–IMS) system (G.A.S. Gesellschaft für analytische Sensorsysteme mbH, Dortmund, Germany) equipped with a PAL3RSI Series II static-headspace autosampler (CTC Analytics AG, Zwingen, Switzerland). A homologous ketone standard mixture containing 2-butanone, 2-pentanone, 2-hexanone, 2-heptanone, 2-octanone, and 2-nonanone was used for retention-index calibration. High-purity nitrogen (≥99.999%) served as both the carrier and drift gas. Samples were analyzed in 20-mL headspace vials using an MXT-WAX capillary column (30 m × 0.53 mm i.d., 1.0 μm film thickness; Restek Corporation, Bellefonte, PA, USA). All samples were rapidly frozen in liquid nitrogen immediately after collection and stored at −80 °C; before analysis, they were thawed in an ice-water bath. After thawing, 1.5 g of muscle was weighed into a headspace vial, sealed, and incubated at 100 °C for 20 min. Headspace conditions were as follows: incubation temperature, 100 °C; incubation time, 20 min; agitation speed, 500 rpm; injection volume, 500 μL; splitless injection; and syringe temperature, 105 °C. The GC column temperature was 60 °C. Carrier-gas flow was maintained at 2.00 mL/min for 2 min, increased linearly to 10.00 mL/min over 8 min, then increased linearly to 100.00 mL/min over 10 min and held for 10 min. The total chromatographic acquisition time was 30 min, and the inlet temperature was 105 °C. IMS conditions were as follows: tritium (^3^H) ionization source, 53-mm drift tube, 2700-V drift voltage, 45 °C drift-tube temperature, nitrogen drift gas (≥99.999%) at 75 mL/min, and positive-ion mode. Each biological sample was analyzed in five technical replicate injections, and the mean peak volume was used for subsequent analyses (Data S1).

Spectra were processed using VOCal 0.4.03 software (G.A.S., Dortmund, Germany). A mixed standard solution of 2-butanone, 2-pentanone, 2-hexanone, 2-heptanone, 2-octanone, and 2-nonanone was used to establish the relationship between retention time and retention index. Target signals were tentatively identified by matching retention indices and relative ion-mobility drift times against the National Institute of Standards and Technology (NIST) 2020 retention-index database and the IMS drift-time database embedded in VOCal. Reporter and Gallery Plot modules were used to display spectra and fingerprint characteristics. Monomer (M) and dimer (D) peaks assigned to the same tentative compound were retained separately for exploratory signal-level analysis to preserve their individual instrumental responses, although these signals may be correlated and do not represent independent chemical compounds. For descriptive chemical-class analysis, the corresponding M and D peak volumes were summed before aggregation by chemical class. For tentatively identified volatile compounds, the compound name or Chemical Abstracts Service (CAS) number was used to search the LRI & Odour Database (http://www.odour.org.uk, accessed on 22 March 2026), Flavornet and Human Odor Space (http://www.flavornet.org, accessed on 22 March 2026), FEMA Flavor Ingredient Library (https://www.femaflavor.org/flavor-library, accessed on 22 March 2026), and The Good Scents Company Information System (http://thegoodscentscompany.com, accessed on 22 March 2026). Published odor descriptions were compiled only to provide chemical context for the candidate signals; they were not used to confirm compound identity and do not imply that sensory evaluation was performed in this study. R 4.4.1 (R Foundation for Statistical Computing, Vienna, Austria) was used to generate the hierarchical-clustering heatmap, principal-component analysis (PCA), stacked plots, volcano plots, and boxplots of candidate GC–IMS signals. Origin 2024 (OriginLab Corporation, Northampton, MA, USA) was used to generate the Pearson-correlation heatmap between the four hydrolyzed amino acids that differed among groups and the candidate GC–IMS signals.

### 2.9. Statistical Analysis

Raw data were organized in Microsoft Excel 2019 (Microsoft Corporation, Redmond, WA, USA), and statistical analyses were performed using IBM SPSS Statistics for Windows 27.0 (IBM Corp., Armonk, NY, USA). Each dietary treatment was represented by one pen containing 10 lambs. Thus, a pen was the unit of dietary treatment allocation, whereas individual lambs were observational units (*n* = 10 for plasma variables and *n* = 6 for postmortem measurements). Because dietary treatments were completely confounded with pens, animal-level comparisons were considered exploratory and could not provide confirmatory evidence of dietary treatment effects. Because no independent pen-level replication was available, no pen-level error term could be estimated for a valid statistical test of dietary treatment effects. Technical replicates from the same biological sample were averaged before statistical analysis. Normality was assessed using the Shapiro–Wilk test together with residual Q–Q plots, and homogeneity of variance was evaluated using the median-based Levene test. For exploratory animal-level comparisons, variables satisfying normality and homoscedasticity assumptions were analyzed by one-way ANOVA across the WS, CSH, and FCSH groups, followed by Duncan’s multiple range test for pairwise comparisons. Variables that were approximately normally distributed but heteroscedastic were analyzed by Welch ANOVA followed by Games–Howell post hoc comparisons. Variables that clearly failed to satisfy parametric assumptions were analyzed by the Kruskal–Wallis test, with Dunn’s test and Holm adjustment used for pairwise comparisons when required (Table S4). Before statistical analysis, suspicious records and potentially influential observations were checked against the original records; observations were not automatically removed solely because they were extreme or affected statistical significance. Results are presented as mean ± standard deviation. Nominal *p* values < 0.05 were used to describe exploratory animal-level differences rather than independently replicated dietary treatment effects. For the 17 individual hydrolyzed amino acids, the omnibus animal-level *p* values (16 one-way ANOVA tests and one Kruskal–Wallis test for cysteine) were adjusted as one testing family using the Benjamini–Hochberg false discovery rate (FDR) procedure. The adjusted values are reported as *q* values, with *q* < 0.05 used as an exploratory screening threshold; the four summary indices (TAA, EAA, NEAA + CEAA, and EAA/TAA) were not included in this correction. For GC–IMS data, 66 instrumental signals, including separately recorded monomer and dimer peaks, were analyzed using exploratory one-way ANOVA. The resulting *p* values were adjusted using the Benjamini–Hochberg false discovery rate (FDR) procedure, with *q* < 0.05 as the adjusted threshold. Separate analysis of potentially correlated M and D signals increased the number of tests and could introduce dependence among features. Therefore, an additional sensitivity analysis was conducted by summing 19 matched M/D pairs within each sample and retaining 28 unmatched signals, yielding 47 features that were analyzed using exploratory one-way ANOVA with Benjamini–Hochberg FDR correction. Fisher’s least significant difference test was used for exploratory pairwise comparisons, but the unadjusted results were not considered confirmatory. Pearson correlations between hydrolyzed amino acids and GC–IMS signals were calculated using animal-matched data and adjusted for multiple testing using the Benjamini–Hochberg method.

## 3. Results

### 3.1. Plasma Biochemical Indices

In exploratory animal-level analyses, no nominal between-group differences were detected in plasma urea, glucose, ALT, AST, ALT/AST, albumin, globulin, ALB/GLB, total protein, triglycerides, or total cholesterol (all *p* > 0.05) (Table 2).

### 3.2. Plasma Antioxidant Indices

In exploratory animal-level comparisons, no nominal between-group differences were detected in T-AOC, CAT, or MDA (all *p* > 0.05) (Table 3). GSH-Px activity was higher in the FCSH group than in the CSH group (nominal *p* < 0.05), whereas no nominal difference was detected between the FCSH and WS groups. T-SOD activity was higher in both the CSH and FCSH groups than in the WS group (nominal *p* < 0.05), with no nominal difference detected between the CSH and FCSH groups.

### 3.3. Slaughter Performance

Among the six lambs selected for slaughter per group, mean pre-slaughter live weight and carcass weight were numerically highest in FCSH, followed by CSH and WS (Table 4). Exploratory animal-level comparisons yielded nominal *p* < 0.05 for these measurements, but do not establish independently replicated dietary treatment effects. Mean post-exsanguination body weight was numerically higher in CSH and FCSH than in WS (nominal *p* < 0.05 in exploratory comparisons), with no nominal difference detected between CSH and FCSH. No nominal between-group differences were detected in dressing percentage, loin-eye area, backfat thickness, or GR value (all *p* > 0.05).

### 3.4. Organ Weights and Indices

In exploratory animal-level comparisons, no nominal between-group differences were detected in the weights or indices of the heart, lungs, liver, spleen, or kidneys (all *p* > 0.05) (Table 5).

### 3.5. Meat Quality

In exploratory animal-level comparisons, no nominal between-group differences were detected in L*, a*, or b* values at 45 min or 24 h postmortem, pH at 45 min or 24 h postmortem, cooking loss, drip loss, or shear force of the longissimus thoracis (all *p* > 0.05) (Table 6).

### 3.6. Proximate Composition of the Longissimus Thoracis

In exploratory animal-level comparisons, moisture content in the longissimus thoracis was higher in the WS group than in the FCSH group (nominal *p* < 0.05). Fat content was higher in the FCSH group than in the WS group (nominal *p* < 0.05), whereas no nominal difference was detected between the FCSH and CSH groups. The CSH group was not distinguished from either group in the post hoc comparisons. No nominal between-group difference was detected in protein content (Table 7).

### 3.7. Hydrolyzed Amino Acid Composition of the Longissimus Thoracis

In exploratory animal-level comparisons, valine, isoleucine, phenylalanine, and proline showed nominal between-group differences (unadjusted *p* < 0.05). Valine and isoleucine were lower in the FCSH group than in the WS group; phenylalanine was lower in the FCSH group than in both the WS and CSH groups; and proline was lower in both the CSH and FCSH groups than in the WS group. However, none of the 17 individual hydrolyzed amino acids met the Benjamini–Hochberg FDR threshold of *q* < 0.05. No nominal between-group differences were detected in TAA, EAA, NEAA + CEAA, or the EAA/TAA ratio (all *p* > 0.05) (Table 8).

### 3.8. GC–IMS Volatile Profiles of the Longissimus Thoracis

**Figure 1:**
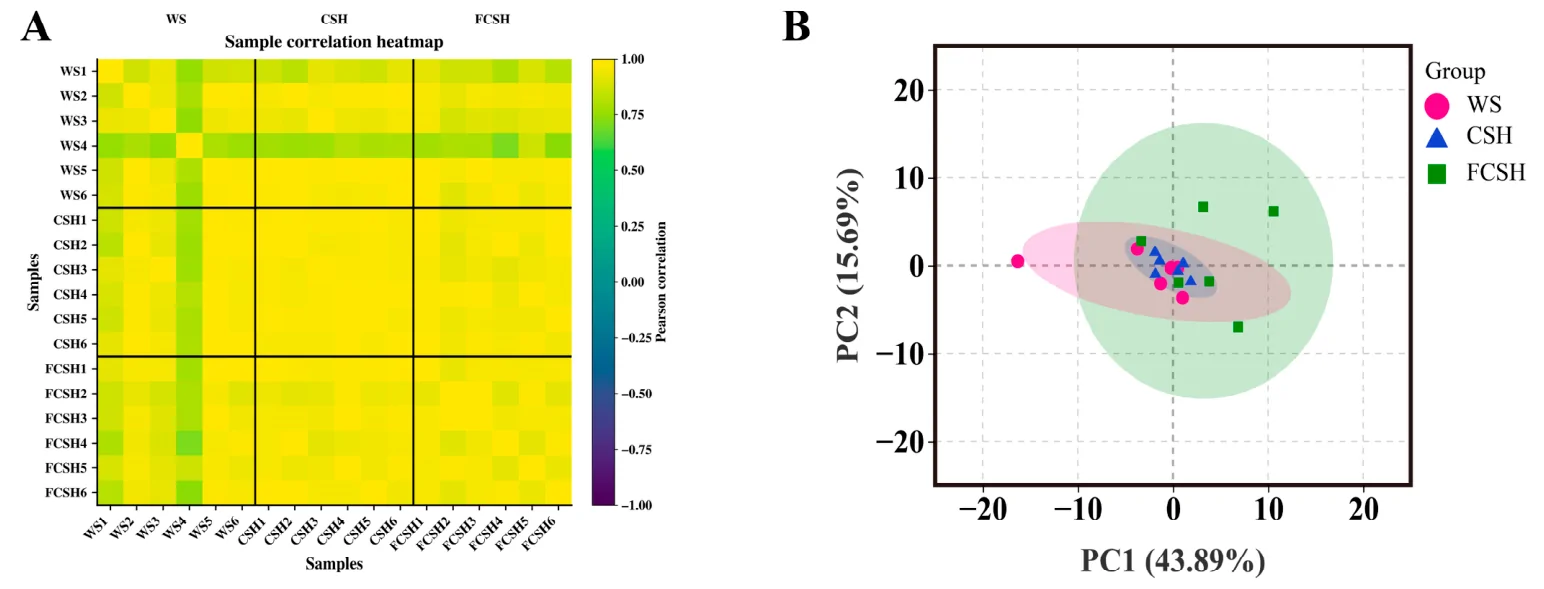
Sample-correlation heatmap and principal component analysis score plot. Note: (**A**) Pearson correlation heatmap based on peak volumes, with samples from the same group arranged adjacently. The color scale in panel (**A**) represents the Pearson correlation coefficients, with yellow indicating stronger positive correlations and purple indicating lower correlation coefficients; (**B**) PCA score plot based on unit-variance-standardized data. Principal component analysis (PCA) was performed on the 66 GC–IMS peak-volume signals from 18 longissimus thoracis samples using unit variance scaling (UV), whereby each signal was mean-centered and divided by its standard deviation before PCA, to visualize the overall distribution of volatile signals among treatment groups. The ellipses represent the corresponding 95% confidence regions for each group. PCA was used for exploratory visualization only, and the confidence ellipses do not establish validated group discrimination.

**Figure 2:**
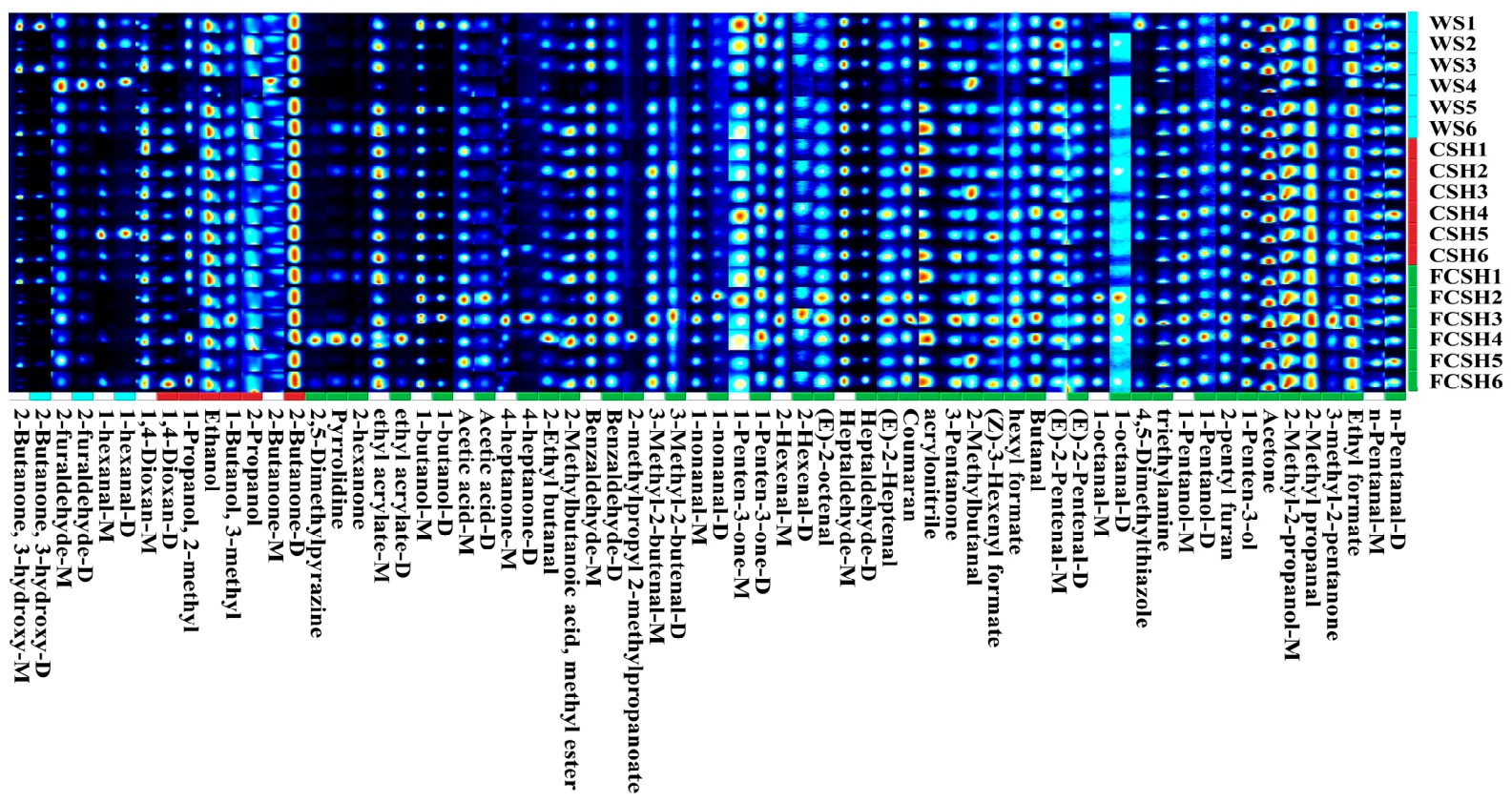
GC–IMS fingerprint of volatile signals in the longissimus thoracis of lambs. Note: Each row represents one sample and each column one volatile signal peak. Spot color and brightness reflect relative signal intensity, with dark blue indicating lower intensity and yellow to red indicating higher intensity. M and D denote monomer and dimer peaks, respectively.

The sample-correlation heatmap showed generally high within-group correlations (Figure 1A), indicating similar overall GC–IMS profiles within groups. PCA based on unit-variance-standardized data (Figure 1B) showed that PC1 and PC2 explained 43.89% and 15.69% of the variance, respectively, and WS showed a tendency to separate along PC1, but this pattern was not supported by confirmatory multivariate testing. The fingerprint plot (Figure 2) showed that most volatile signals were detected in all three groups. Samples within the same group had similar fingerprints, while the intensities of several characteristic peaks differed among groups.

**Figure 3:**
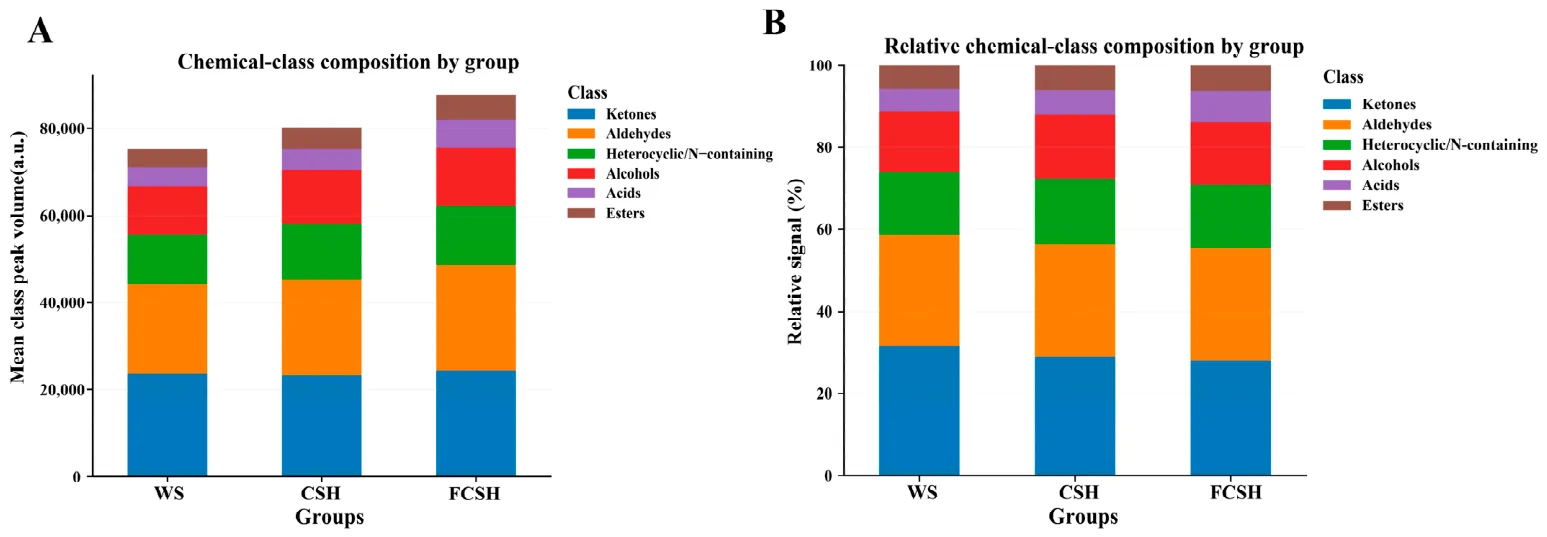
Stacked plots of tentatively assigned GC–IMS volatile-signal classes in the longissimus thoracis of Kazakh lambs. Note: (**A**) Summed peak-volume signals by chemical class; (**B**) relative signal percentage of each class. For class-composition analysis, M and D peak volumes from the same compound were first summed and then grouped by chemical class. Panel A shows the mean class peak volume for each treatment, and the relative plot shows the percentage of each class in the total peak volume of that treatment.

**Figure 4:**
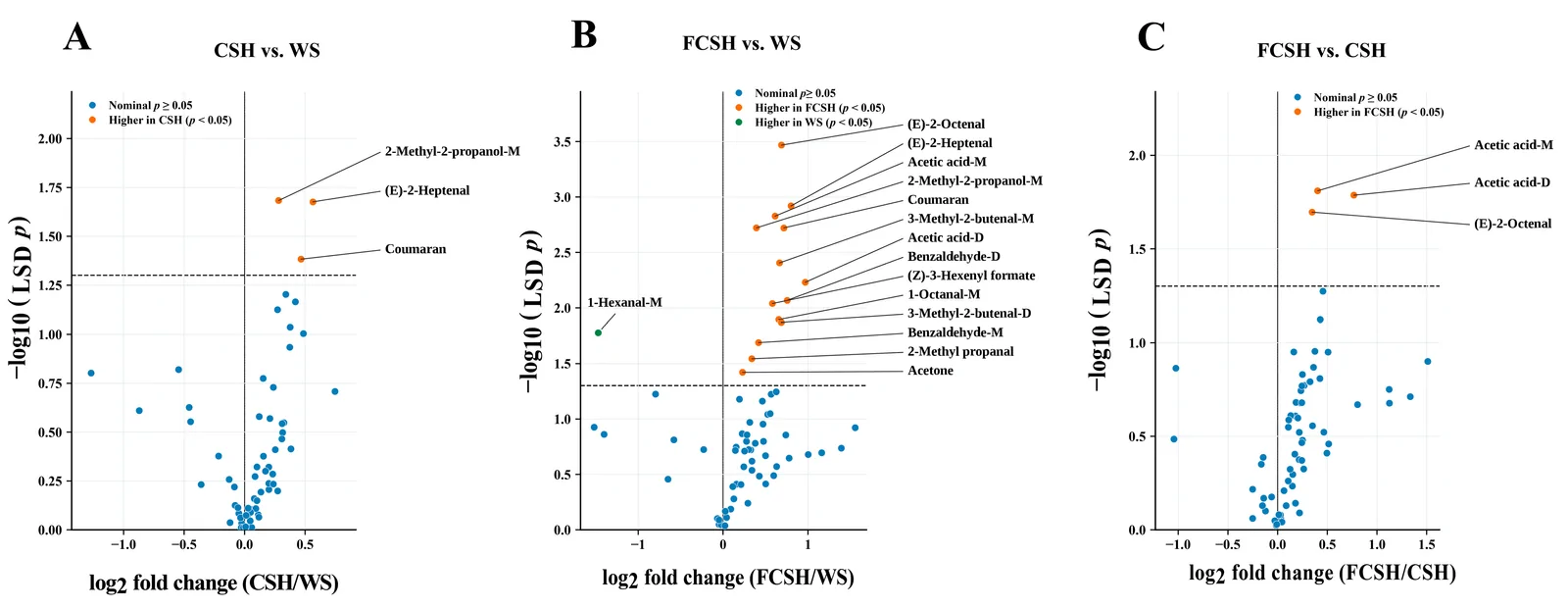
Pairwise volcano plots of GC–IMS volatile signals. Note: (**A**) CSH group vs. WS group; (**B**) FCSH group vs. WS group; (**C**) FCSH group vs. CSH group. The x-axis represents log_2_ fold change based on mean peak volumes, and the y-axis represents −log_10_(unadjusted Fisher’s LSD *p* value). Orange and green indicate exploratory candidate signals with unadjusted *p* < 0.05 and relatively higher or lower peak volumes, respectively; blue indicates *p >* 0.05. These pairwise comparisons were not adjusted for multiple testing and are presented for exploratory screening only.

**Figure 5:**
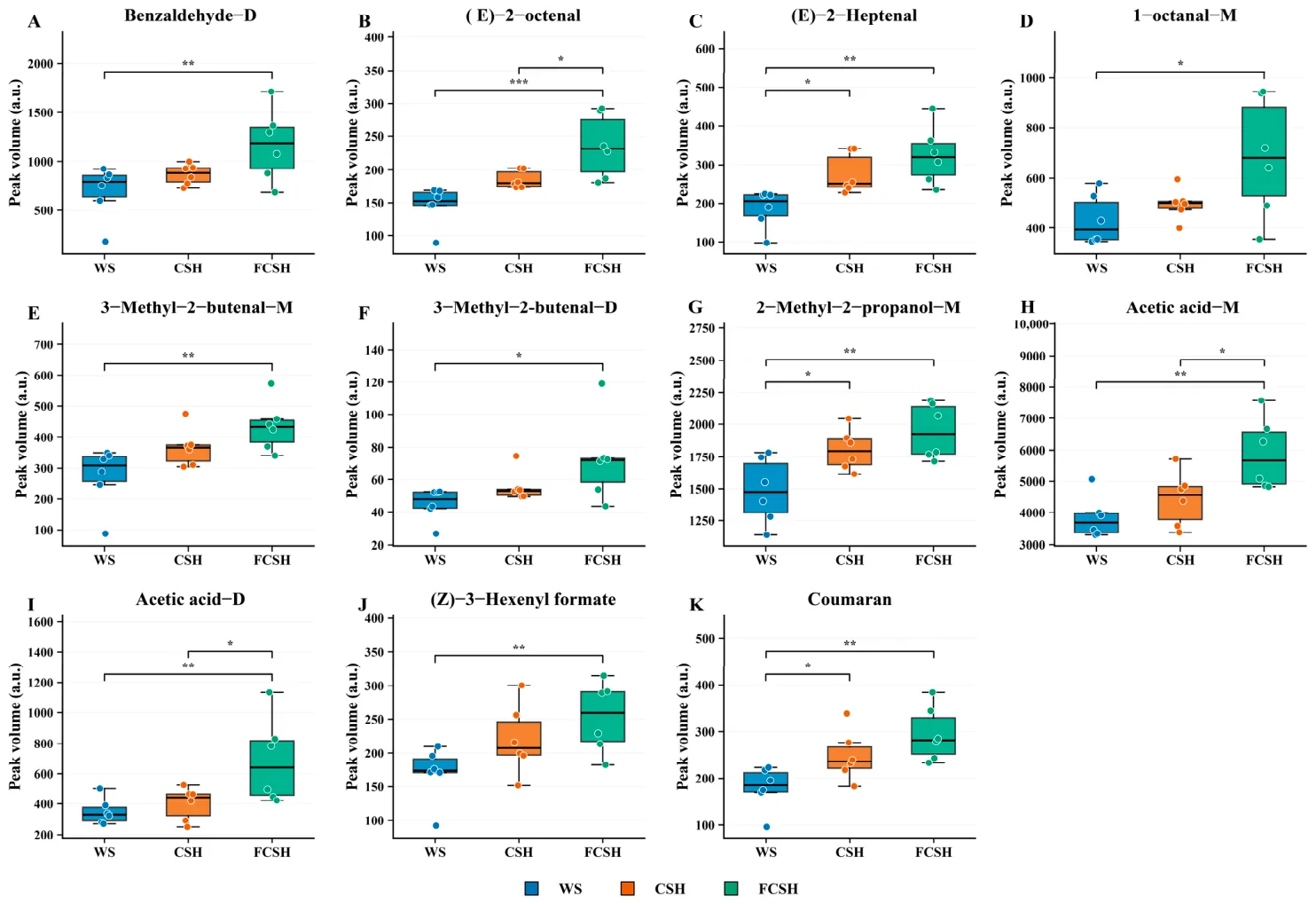
GC–IMS candidate signals with nominal *p* < 0.05 in one-way ANOVA before FDR correction. Note: Signals with unadjusted omnibus ANOVA *p* < 0.05 are displayed, and brackets with asterisks indicate unadjusted Fisher’s LSD pairwise comparisons. These pairwise comparisons are exploratory and do not represent FDR-adjusted significance. (**A**) Benzaldehyde-D; (**B**) (E)-2-octenal; (**C**) (E)-2-heptenal; (**D**) 1-octanal-M; (**E**) 3-methyl-2-butenal-M; (**F**) 3-methyl-2-butenal-D; (**G**) 2-methyl-2-propanol-M; (**H**) acetic acid-M; (**I**) acetic acid-D; (**J**) (Z)-3-hexenyl formate; (**K**) coumaran. Asterisks indicate unadjusted pairwise comparisons based on Fisher’s LSD test (* *p* < 0.05, ** *p* < 0.01, and *** *p* < 0.001) and are presented for exploratory purposes only. These pairwise *p* values were not adjusted for multiple comparisons. After Benjamini–Hochberg FDR correction across the 66 GC–IMS signals, none remained significant at *q* < 0.05.

**Figure 6:**
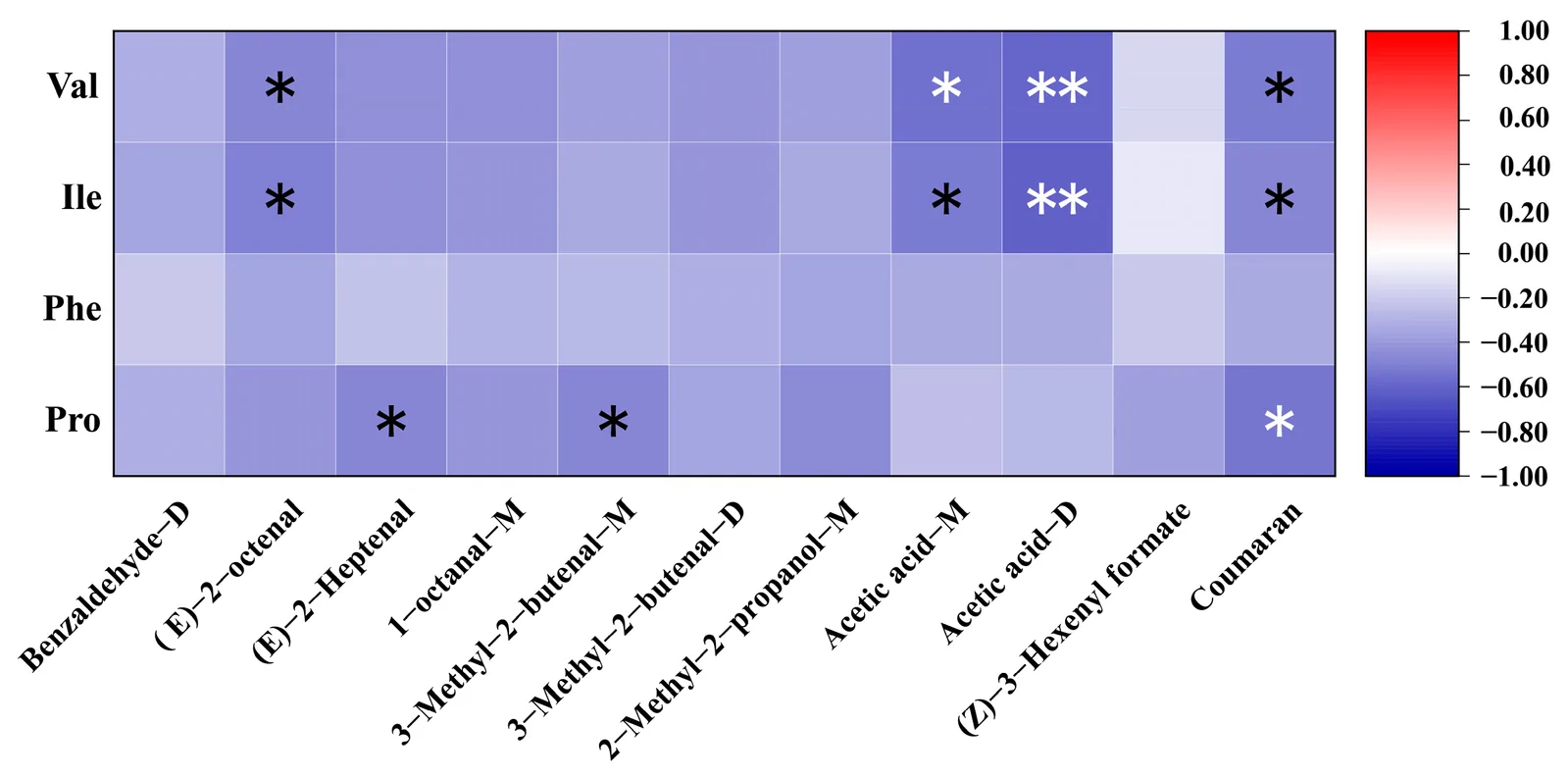
Pearson correlation heatmap between hydrolyzed amino acids and GC–IMS volatile signals. Note: The color scale represents Pearson’s correlation coefficient (r), indicating the direction and magnitude of the associations. Asterisks indicate nominal statistical significance before multiple-testing correction (* *p* < 0.05, ** *p* < 0.01); correlations without asterisks are not significant at the unadjusted *p* < 0.05 level. After Benjamini–Hochberg FDR correction, none of the tested correlations reached *q* < 0.05.

The chemical-class composition of volatile signals and the pairwise volcano plots are presented in Figure 3 and Figure 4. Mean acid and aldehyde signal intensities were numerically higher in the FCSH group than in the WS and CSH groups (Figure 3A), whereas their relative proportions were broadly similar among the three groups (Figure 3B). Exploratory volcano plots based on unadjusted Fisher’s LSD *p* values identified 3 higher candidate signals for the CSH group vs. WS group, 14 higher and 1 lower candidate signal for the FCSH group vs. WS group, and 3 higher candidate signals for the FCSH group vs. CSH group at the nominal *p* < 0.05 threshold. Across the 66 GC–IMS signals, 11 had an unadjusted omnibus ANOVA *p* < 0.05 and are displayed in Figure 5. In unadjusted pairwise comparisons, the FCSH group showed nominally higher benzaldehyde-D, (E)-2-octenal, (E)-2-heptenal, 1-octanal-M, 3-methyl-2-butenal-M, and 3-methyl-2-butenal-D signals than the WS group (*p* < 0.05). The signal tentatively assigned to (E)-2-octenal was nominally higher in the FCSH group than in both the WS and CSH groups, whereas the signal tentatively assigned to (E)-2-heptenal was nominally higher in the CSH and FCSH groups than in the WS group (unadjusted *p* < 0.05). Among alcohols, 2-methyl-2-propanol-M was nominally higher in the CSH and FCSH groups than in the WS group. Among acids, acetic acid-M and acetic acid-D were nominally higher in the FCSH group than in both the WS and CSH groups, whereas the WS and CSH groups did not differ. Among esters, (Z)-3-hexenyl formate was nominally higher in the FCSH group than in the WS group. Among heterocyclic compounds, coumaran was nominally higher in the CSH and FCSH groups than in the WS group, with no difference between the CSH and FCSH groups. After Benjamini–Hochberg FDR correction, none of the 66 GC–IMS signals reached *q* < 0.05 (Table S1); therefore, the above signals are interpreted only as exploratory candidates. The M/D-combined sensitivity analysis reduced the number of features to 47, of which 9 showed nominal differences (unadjusted *p* < 0.05), but none reached *q* < 0.05 after FDR correction (Table S2). Pearson correlations were subsequently calculated between four hydrolyzed amino acids that differed among groups and the 11 candidate GC–IMS signals using the 18 muscle samples (Figure 6). Before correction, Val and Ile showed negative correlations with the GC–IMS signals assigned to (E)-2-octenal, acetic acid-M, acetic acid-D, and coumaran (unadjusted *p* < 0.05), with the correlation involving acetic acid-D reaching unadjusted *p* < 0.01. After BH-FDR correction, none of these correlations remained significant (Table S3).

## 4. Discussion

### 4.1. Plasma Biochemical and Antioxidant Indices

In this study, no nominal between-group differences were detected in the measured plasma biochemical indices, including urea and glucose, among animals assigned to the WS, CSH, and FCSH diets. However, these animal-level comparisons do not establish the absence of dietary effects because each diet was represented by only one pen. Zanine et al. [3] included 0%, 10%, 20%, and 30% CSH in ram diets and found no significant effects on plasma urea or glucose concentrations. Magalhães et al. [30] reported that most serum metabolic indices and the activities of ALT, AST, and GGT were unchanged across cottonseed-hull/chitosan treatments; serum cholesterol decreased only when chitosan was combined with whole CSH. These findings are broadly consistent with the absence of detectable between-group differences in the measured plasma biochemical indices in the present study. Previous studies have suggested that ruminal microorganisms may contribute to the degradation of residual free gossypol in cottonseed products [4,31,32]; however, ruminal detoxification was not directly assessed in the present study. In the present study, plasma GSH-Px activity was nominally higher in animals assigned to the FCSH group than in those assigned to the CSH group, while T-SOD activity was nominally higher in both the CSH and FCSH groups than in the WS group, with no nominal difference detected between CSH and FCSH. T-AOC, CAT, and MDA showed no nominal between-group differences. The higher T-SOD activity observed in both CSH and FCSH relative to WS was not specific to the fermented cottonseed-hull diet. By contrast, GSH-Px activity was higher in FCSH than in CSH but was not detectably different from WS. Thus, the observed GSH-Px difference is compatible with variation in glutathione-dependent antioxidant-enzyme responses among the study groups, but it cannot be attributed specifically to fermentation because dietary treatment was confounded with pen. SOD mainly catalyzes dismutation of superoxide anions, whereas GSH-Px removes peroxides through the glutathione system. The higher T-SOD in both CSH and FCSH, together with a GSH-Px difference only between FCSH and CSH, may therefore be consistent with enzyme-specific variation, although the contributions of dietary composition, fermentation products, and pen-related factors cannot be distinguished in this study. Previous research has shown that postbiotics derived from *Lactiplantibacillus plantarum* RG14 significantly increased GPx activity in serum and rumen fluid of post-weaning lambs and upregulated hepatic *GPX1*, *GPX4*, and *Cu/Zn-SOD* expression [33], suggesting that *L. plantarum* or its metabolites may regulate antioxidant enzymes in ruminants. Our previous untargeted metabolomics study further showed that compound fermentation with *Aspergillus niger*, *Aspergillus oryzae*, and *Lactiplantibacillus plantarum* markedly altered the small-molecule metabolite profile of CSH and enriched metabolites such as L-arginine and trans-ferulic acid. Zhang et al. [34] reported that supplementation with 1% L-arginine in intrauterine growth-retarded lambs significantly increased hepatic SOD, GSH-Px, and GSH and decreased MDA. Thus, increases in arginine and related metabolites reported after fermentation may provide biological context for the observed antioxidant-enzyme patterns; however, their contributions to the present findings remain hypothetical because the relevant metabolite exposure and its relationship with plasma enzyme activities were not directly evaluated. Li et al. [35] fed lactating goats alfalfa silage inoculated with ferulic-acid-esterase-producing *Limosilactobacillus reuteri* A4-2 and found that the treatment increased T-AOC, SOD, and GPx activities in the silage itself and also increased serum T-AOC, SOD, GPx, and CAT activities. Another study using alfalfa silage fermented with the highly antioxidant *Lactiplantibacillus plantarum* 24-7 similarly showed increased serum SOD, GPx, CAT, and T-AOC in lactating goats [36]. These studies provide possible biological context for the observed antioxidant-enzyme patterns, although the contributions of individual microorganisms and fermentation-derived metabolites were not directly evaluated in the present study. Studies of *Aspergillus oryzae* culture in sheep have also shown increased accumulation of antioxidant-related metabolites, including lipoamide, in rumen fluid [37]. In addition, supplementation of periparturient buffaloes with 5 g of *Aspergillus oryzae* per head per day significantly increased plasma CAT and GPx, decreased MDA, and reduced inflammatory markers including haptoglobin and TNF-α [38]. These studies provide a possible biological context for the difference in GSH-Px activity between FCSH and CSH, but they do not establish that these microorganisms or metabolites contributed to the differences observed in the present experiment. Accordingly, the findings should be interpreted as exploratory animal-level differences in selected antioxidant-enzyme activities among the three diet-assigned pens, rather than as evidence that fermentation improved overall antioxidant capacity or produced a reproducible dietary treatment effect.

### 4.2. Slaughter Performance and Organ Indices

Slaughter traits are relevant to production outcomes in meat animals. Among the six lambs selected for slaughter in each group, animals assigned to FCSH showed nominally higher pre-slaughter live weight and absolute carcass weight than those assigned to CSH or WS; the CSH subset also showed nominally higher values for both measurements than the WS subset. Post-exsanguination body weight was nominally higher in the CSH and FCSH subsets than in the WS subset, with no nominal difference detected between CSH and FCSH. However, no nominal between-group differences were detected in dressing percentage, loin-eye area, backfat thickness, or GR value. Thus, the observed differences concerned absolute body weight at slaughter and absolute carcass weight, rather than proportional carcass yield or major carcass-conformation measurements. In particular, a higher absolute carcass weight accompanying higher live weight cannot establish improved feed conversion or proportional saleable yield. Ren et al. [9] found no significant difference in dressing percentage when FCSH replaced corn silage in finishing Kazakh sheep. Yusuf et al. [10] likewise reported no significant changes in dressing percentage or loin-eye area in Hu sheep receiving a fermented total mixed ration containing cottonseed meal. These studies provide contextual support for considering absolute weight separately from carcass-yield indices, but the present comparisons remain exploratory. *Aspergillus oryzae* culture and microbially fermented roughages have been associated with changes in fiber degradation and rumen-fermentation characteristics [37]. Such mechanisms may be relevant hypotheses, but the present study did not report individual dry matter intake, feed conversion, nutrient digestibility, or rumen-fermentation measurements. Moreover, because formal-period average daily gain and baseline-adjusted endpoint analyses were not reported, the higher observed slaughter-endpoint weights should not be attributed specifically to dietary fermentation or interpreted as evidence of enhanced feed efficiency. The measured organ weights and relative indices of the heart, liver, lungs, spleen, and kidneys showed no nominal between-group differences. The slaughter-related findings apply only to the selected six-lamb subsets and cannot disentangle dietary treatment from pen-related factors.

### 4.3. Meat Quality, Proximate Composition, and Hydrolyzed Amino Acids

In this study, no nominal between-group differences were detected in L*, a*, and b* values and pH measured at 45 min and 24 h postmortem, or in cooking loss, drip loss, and shear force. Thus, among the six lambs selected for slaughter per group, the higher absolute carcass weight observed in animals assigned to FCSH was not accompanied by detectable differences in these measured conventional meat-quality traits. However, the absence of statistically detectable differences should not be interpreted as evidence of equivalence among treatments. Yusuf et al. [10] fed Hu sheep a fermented total mixed ration containing cottonseed meal for 97 d and likewise found no significant changes in longissimus thoracis meat color, drip loss, cooking loss, or shear force. Zhou et al. [39] found that supplementation with *Lactiplantibacillus plantarum* N-1 alone did not significantly alter longissimus thoracis meat color, cooking loss, drip loss, or water-holding capacity in Hu sheep; shear force decreased significantly only when *L. plantarum* N-1 was combined with isomaltooligosaccharide. These observations are broadly consistent with the lack of detectable differences in the measured physical meat-quality traits in the present study, although they do not establish that fermented cotton by-products or particular microbial strains have no effect on meat quality. In contrast to the absence of detectable differences in conventional physical meat-quality traits, moisture content in the longissimus thoracis was nominally lower in the FCSH group than in the WS group, whereas crude fat content was nominally higher; the CSH group showed intermediate values for both traits, and no nominal between-group differences were detected in crude protein content. Because no nominal differences in moisture or crude fat were detected between FCSH and CSH, these results are more appropriately interpreted as exploratory animal-level differences in proximate muscle composition between the selected FCSH and WS lambs, rather than as evidence that fermentation itself caused the difference in crude-fat content. Furthermore, because each dietary treatment was represented by a single pen, the contributions of dietary treatment and pen-related factors cannot be separated. Previous studies indicate that fermented-diet composition can affect muscle fat content and fatty acid composition in lambs, potentially through changes in rumen fermentation, nutrient-substrate supply, and lipid-metabolism regulation [12]. However, muscle fatty acid composition and lipid-metabolism-related indicators were not measured in the present study, so these mechanisms are presented only as possible biological context for the current findings. The higher pre-slaughter live weight observed among the slaughter-selected lambs assigned to FCSH should also be considered when interpreting differences in muscle crude fat. Rant and Radzik-Rant [40] reported that slaughter weight can influence intramuscular fat content in some muscles, with higher slaughter weight being accompanied by higher intramuscular fat in some breeds. Thus, the crude-fat difference observed here may also be associated with body-weight status at slaughter, and its specific attribution to dietary fermentation is not supported by the present experimental design. Notably, the higher crude fat content in the FCSH group was not accompanied by detectable between-group differences in drip loss, cooking loss, or shear force. Zhang et al. [41] likewise found in 76 Tan sheep that intramuscular fat content was not significantly correlated with drip loss, cooking loss, or shear force. Hydrolyzed amino acid analysis revealed no nominal between-group differences in TAA, EAA, or EAA/TAA. Deng [42] found that fermented total mixed rations containing cotton by-products did not significantly alter TAA or EAA contents in the longissimus thoracis of lambs, while EAA/TAA also remained stable. Similarly, Yusuf et al. [10] reported that fermented total mixed rations containing cottonseed meal altered only some amino acid contents. These findings are broadly consistent with the present results, in which no nominal differences were detected in the overall amino acid summary measures. Although four individual hydrolyzed amino acids showed nominal between-group differences, none remained statistically significant after Benjamini–Hochberg FDR correction across the 17 individual amino acid tests (all *q* ≥ 0.05). Therefore, these individual amino acid differences should be interpreted as exploratory observations rather than confirmed dietary effects. Moreover, the present findings on meat quality, proximate composition, and hydrolyzed amino acids apply specifically to the slaughter-selected subset and should not be directly generalized to all animals initially assigned to each dietary treatment.

### 4.4. GC–IMS Volatile Profiles

Volatile compounds are an important basis of lamb flavor and are influenced by diet, muscle lipid deposition, amino acid composition, and heating conditions. The GC–IMS fingerprints in this study showed that most volatile signals were detected across the WS, CSH, and FCSH groups, whereas some signals displayed different between-group peak-volume patterns; PCA visually suggested some separation in the overall volatile-signal profiles of the three groups; this was a descriptive pattern and was not supported by confirmatory multivariate testing. Wang et al. [43] similarly used GC–IMS to analyze different Chinese indigenous sheep breeds and found that lamb volatiles were composed mainly of aldehydes, alcohols, ketones, esters, and acids, with sample differences expressed primarily as changes in the intensities of selected volatile signals and in overall fingerprint patterns. Thus, the observed patterns mainly reflect nominal variation in selected volatile signals rather than a broad change in the overall GC–IMS fingerprint. Wang et al. [14] further showed that GC–IMS combined with PCA can effectively characterize differences in volatile fingerprints among lamb samples. In the present study, one-way ANOVA of all 66 GC–IMS signals identified 11 signals with unadjusted omnibus *p* < 0.05, involving mainly aldehydes, acetic acid, alcohols, esters, and other volatile signals. In subsequent pairwise comparisons, the FCSH group showed nominally higher peak volumes for several aldehydes and acetic-acid M/D signals relative to WS, and some signals also showed nominal between-group differences between FCSH and CSH. These candidate signals warrant further investigation. Because 66 signals were tested simultaneously, Benjamini–Hochberg FDR correction was applied; none of these candidate signals reached *q* < 0.05. They are therefore interpreted as exploratory candidates for subsequent biological interpretation and validation, while Fisher’s LSD results in Figure 4 and Figure 5 are used mainly to describe possible directions of change. Aldehydes were among the volatile classes with the most concentrated changes. Lipids and their fatty acid composition are important precursors of volatile aldehydes in meat. Li et al. [15] compared Hu sheep with high and low intramuscular fat using lipidomics and volatilomics and found marked differences in triglyceride and diglyceride composition accompanied by corresponding differences in volatile aroma profiles; lipid-derived compounds were considered major contributors to the aroma differences, and thermal oxidation of unsaturated fatty acids may have participated in this process. More recently, Yang et al. [44] further showed from the perspective of lamb lipid oxidation that oxidation altered the muscle lipid profile and was accompanied by increases in multiple volatile aldehydes, further supporting a close link between lipid oxidation and volatile-aldehyde formation in lamb. Although lipid oxidation during heating may contribute to aldehyde-related signals, including the signal tentatively assigned to (E)-2-octenal, the present data do not establish their specific formation pathways or demonstrate that the observed differences resulted from feed fermentation. Dietary microbial fermentation may also participate indirectly in the formation of volatile precursors by altering rumen fermentation and downstream lipid metabolism. Our previous work showed that compound solid-state fermentation with *Aspergillus niger*, *Aspergillus oryzae*, and *Lactiplantibacillus plantarum* reduced NDF and ADF in cottonseed hulls and altered their small-molecule metabolite composition [18]. Xie et al. [37] further found that *Aspergillus oryzae* culture improved fiber utilization and altered rumen-fermentation characteristics in sheep. Notably, Li et al. [13] found that dietary yeast culture not only increased total ruminal VFA and acetate concentrations, but was also accompanied by increased intramuscular fat and expression of lipid-metabolism-related genes such as PPAR, FASN, and FABP4, together with changes in multiple volatiles including hexanal, nonanal, and benzaldehyde. These studies provide possible mechanistic context, although rumen fermentation, muscle fatty acid composition, and lipid-metabolism pathways were not directly evaluated in the present study. In addition to lipids, volatile compounds are affected by multiple precursor classes. Wang et al. [14] found that fat, fatty acids, free amino acids, and other aroma precursors were closely related to volatile compounds in cooked lamb, and aldehydes including heptanal, hexanal, octanal, and nonanal differed markedly among meat samples. This indicates that meat volatile profiles generally arise from the combined effects of multiple precursors and thermal reactions. In the present study, hydrolyzed amino acids also showed some correlations with selected GC–IMS candidate signals before adjustment, but none met the significance criterion after BH-FDR correction; moreover, the present study measured hydrolyzed rather than free amino acids. It should be emphasized that the acid-hydrolysis method used here measures amino acid composition after hydrolysis of muscle proteins, rather than the free-amino-acid pool in muscle. A recent review of taste-active compounds in meat noted that the amino acids directly involved in taste formation are primarily free amino acids, together with taste-active peptides, nucleotides, organic acids, and other soluble substances [45]. Therefore, these correlations are better viewed as leads for future studies of free amino acids and other flavor precursors.

The GC–IMS analysis was conducted after headspace incubation at 100 °C for 20 min; thus, the results reflect volatile signals under a standardized heating condition. In particular, acetic-acid signals detected in heated meat headspace cannot be used to infer ruminal acetate production or lipid synthesis. Heating itself is an important determinant of meat volatiles. Wu et al. [46] used HS-GC–IMS in 2025 to study volatile changes in Sonit lamb during different grilling times, detecting 83 volatile compounds and observing marked changes in characteristic signals such as (E)-2-octenal and nonanal as heating time changed, indicating that time and temperature can substantially shape lamb volatile fingerprints. Accordingly, the GC–IMS profile obtained here at 100 °C for 20 min should be regarded as a volatile fingerprint under a standardized heating condition, which also ensures comparable analytical conditions among the three treatment groups. Sensory evaluation was not performed in the present study; therefore, typical odor descriptions of compounds in the Supplementary Materials are intended only to provide chemical context for candidate signals and do not directly demonstrate corresponding sensory attributes in the samples. Overall, the results suggest that FCSH may be associated with nominal between-group differences in selected aldehyde, acid, and other signals in the lamb volatile fingerprint, providing candidate patterns for further mechanistic investigation. These observations support further study of FCSH as an unconventional roughage substitute. Under the conditions of the present experiment, animals in the FCSH group showed greater absolute carcass weight, whereas dressing percentage and major conventional meat-quality traits did not differ markedly among groups. This may be of practical relevance for utilizing cotton-processing by-products and expanding unconventional roughage resources in major cotton-producing regions. Because each diet was represented by only one pen, these findings require confirmation in studies with independent pen replication.

### 4.5. Study Limitations

This study has several experimental-design limitations. First, the three diets each corresponded to a single independent pen, with only one pen per diet. Consequently, dietary treatment and pen effects cannot be separated, and within-treatment variation among independent pens cannot be estimated reliably. Although plasma measurements were obtained from individual lambs and carcass, meat-quality, hydrolyzed-amino-acid, and volatile-compound measurements were obtained from selected slaughtered lambs, these individual observations cannot be regarded as independent dietary-treatment replicates. Accordingly, the animal-level between-group comparisons in this study are exploratory and require validation in studies with independent pen replication. Second, GC–IMS provides volatile-signal-profile information, and compound identification based on database matching is tentative; sensory evaluation was not conducted. Therefore, differences in volatile signals cannot be used directly to infer actual sensory changes in meat flavor. Coded samples and partial blinding were implemented during laboratory measurements, but complete blinding throughout the animal nutrition trial was not feasible because dietary treatments involved practical feeding management. Treatment allocation may therefore have been known during animal management or some experimental procedures and could represent a potential source of bias. Future studies could use full-process sample coding and independent data-analysis workflows to further reduce such bias. The slaughter subset comprised six lambs per group selected according to the prespecified procedure described above. However, excluding the animals with the highest and lowest body weights may have reduced within-group variability and introduced selection bias. Nevertheless, because carcass and muscle measurements were obtained from six rather than all ten lambs per group, these outcomes specifically reflect the slaughter-sampled subset and should not be generalized to all ten lambs initially assigned to each dietary treatment. Growth-performance and feed-utilization outcomes from the feeding trial are not reported in this manuscript, which focuses on carcass and meat characteristics. Therefore, differences in slaughter-endpoint weight cannot be interpreted as evidence of improved growth performance, feed efficiency, or nutrient utilization. In addition, the higher muscle fat content in the FCSH group occurred together with changes in several unadjusted GC–IMS candidate signals, providing a lead for further study of potential links between muscle lipid composition and volatile precursors. Future work should further investigate the mechanisms underlying differences in muscle fat content among roughage treatments and may integrate analyses of free amino acids, free fatty acids, quantitative volatile compounds, and sensory evaluation to clarify associations among FCSH feeding, muscle composition, volatile profiles, and sensory outcomes and to provide stronger evidence for its rational use in lamb production.

## 5. Conclusions

Under the conditions of this study, no nominal between-group differences were detected in conventional plasma biochemical indices or the measured longissimus thoracis color, pH, cooking loss, drip loss, and shear force among the WS, CSH, and FCSH groups. Among the six lambs selected for slaughter per group, those assigned to the FCSH diet had nominally higher pre-slaughter live weight and absolute carcass weight than those in the WS and CSH groups. Plasma GSH-Px activity was nominally higher in FCSH than in CSH but not WS, while T-SOD activity was nominally higher in both CSH and FCSH than in WS. Crude fat content in the longissimus thoracis was nominally higher in FCSH than in WS, but no nominal difference was detected between FCSH and CSH. Although four individual hydrolyzed amino acids showed nominal between-group differences, none reached *q* < 0.05 after Benjamini–Hochberg FDR correction; no nominal between-group differences were detected in TAA, EAA, or EAA/TAA. Exploratory GC–IMS analysis identified 11 of 66 volatile signals with unadjusted omnibus *p* < 0.05, but none reached *q* < 0.05 after Benjamini–Hochberg FDR correction. These volatile-signal differences therefore remain exploratory and do not establish confirmed changes in meat flavor. Because each diet was represented by only one pen, dietary treatment effects cannot be separated from pen-related factors, and the observed animal-level differences do not establish causal effects of dietary fermentation. Furthermore, carcass and muscle findings are limited to the selected slaughter subsets and cannot be directly generalized to all animals initially assigned to each group. Overall, these observations are preliminary descriptions of lambs in three diet-assigned pens rather than evidence of reproducible dietary treatment effects and require confirmation in studies with independent pen replication.

## Figures and Tables

**Table 1:** Composition and nutrient levels of the experimental diets (dry matter basis).

Ingredient	WS	CSH	FCSH
Wheat straw–wheat bran mixture (77:23)	25	--	--
Unfermented cottonseed hull–wheat bran mixture (80:20)	--	25	--
Fermented cottonseed hull–wheat bran mixture (80:20)	--	--	25
Alfalfa	15	15	15
Corn	36.8	36.8	36.8
Soybean meal	12	12	12
Wheat bran	5.2	5.2	5.2
Expanded urea	1	1	1
NaHCO_3_	1	1	1
Premix	4	4	4
Nutrient levels			
Gross energy, MJ/kg	17.92	18.05	17.90
Crude protein, %	14.27	14.78	14.97
Neutral detergent fiber, %	33.71	37.75	35.42
Acid detergent fiber, %	21.38	24.22	22.27
Crude fat, %	2.58	2.89	2.78
Ca, %	0.94	0.86	0.84
P, %	0.46	0.51	0.49

Note: --, not added. All ingredient proportions are expressed on a dry-matter basis. The replacement mixtures accounted for 25% of each diet and consisted of wheat straw and wheat bran (77:23) in the WS group or cottonseed hulls and wheat bran (80:20) in the CSH and FCSH groups. The mixture used in the FCSH group underwent solid-state fermentation before feeding. Wheat bran added separately is listed independently. The premix provided per kilogram of diet: vitamin A, 4800 IU; vitamin D, 1500 IU; vitamin E (DL-α-tocopheryl acetate), 28 IU; biotin, 0.8 mg; Cu, 6.0 mg; Fe, 16 mg; Mn, 20 mg; organic Mn, 5.2 mg; Zn, 40 mg; organic Zn, 12.8 mg; I, 0.8 mg; Se, 0.28 mg; and Co, 0.4 mg. Nutrient levels were determined by chemical analysis.

**Table 2:** Plasma biochemical indices of Kazakh lambs across dietary groups.

Item	WS	CSH	FCSH	*p* Value
Urea, mmol/L	7.14 ± 0.76	6.97 ± 0.96	7.09 ± 0.89	0.765
Glucose, mmol/L	5.28 ± 0.67	4.94 ± 0.46	4.94 ± 0.66	0.370
ALT, U/L	26.60 ± 9.17	19.20 ± 8.16	22.10 ± 7.31	0.149
AST, U/L	122.60 ± 11.76	120.80 ± 18.72	116.30 ± 19.40	0.697
ALT/AST	0.21 ± 0.06	0.16 ± 0.06	0.19 ± 0.06	0.127
ALB, g/L	36.27 ± 1.39	36.51 ± 1.28	35.62 ± 1.29	0.313
GLB, g/L	29.47 ± 3.24	28.33 ± 2.72	27.32 ± 2.08	0.228
ALB/GLB	1.25 ± 0.15	1.30 ± 0.13	1.31 ± 0.11	0.543
Total protein, g/L	65.74 ± 3.19	64.84 ± 2.79	62.91 ± 2.45	0.091
Triglycerides, mmol/L	0.16 ± 0.06	0.17 ± 0.05	0.12 ± 0.05	0.117
Total cholesterol, mmol/L	1.31 ± 0.18	1.43 ± 0.15	1.37 ± 0.10	0.281

Note: Animal-level *p* values are exploratory. Urea was analyzed using the Kruskal–Wallis test, total cholesterol using Welch ANOVA, and all other variables using one-way ANOVA. ALT, alanine aminotransferase; AST, aspartate aminotransferase; ALB, albumin; GLB, globulin.

**Table 3:** Plasma antioxidant indices of Kazakh lambs across dietary groups.

Item	WS	CSH	FCSH	*p* Value
MDA, nmol/mL	2.23 ± 0.53	1.77 ± 0.48	1.87 ± 0.40	0.090
T-AOC, mmol/L	0.84 ± 0.09	0.83 ± 0.10	0.90 ± 0.09	0.188
CAT, U/mL	0.19 ± 0.08	0.21 ± 0.10	0.20 ± 0.06	0.795
GSH-Px, U/mL	300.13 ± 42.32 ^ab^	275.06 ± 60.12 ^b^	351.91 ± 64.46 ^a^	0.016
T-SOD, U/mL	115.26 ± 16.58 ^b^	136.25 ± 15.86 ^a^	129.78 ± 10.89 ^a^	0.011

Note: Animal-level *p* values and, where applicable, superscript letters are exploratory. All variables were analyzed using one-way ANOVA, followed by Duncan’s multiple range test where appropriate. Different lowercase superscript letters within a row indicate nominal pairwise differences (*p* < 0.05). MDA, malondialdehyde; T-AOC, total antioxidant capacity; CAT, catalase; GSH-Px, glutathione peroxidase; T-SOD, total superoxide dismutase.

**Table 4:** Slaughter performance of Kazakh lambs across dietary groups.

Item	WS	CSH	FCSH	*p* Value
Pre-slaughter live weight, kg	38.4 ± 0.8 ^c^	40.4 ± 1.3 ^b^	42.1 ± 1.0 ^a^	<0.001
Post-exsanguination body weight, kg	37.1 ± 0.9 ^b^	39.1 ± 1.2 ^a^	40.4 ± 1.1 ^a^	<0.001
Carcass weight, kg	20.5 ± 1.0 ^c^	22.5 ± 0.5 ^b^	23.6 ± 0.9 ^a^	<0.001
Dressing percentage, %	53.5 ± 2.7	55.7 ± 1.9	56.1 ± 1.3	0.088
Loin-eye area, cm^2^	17.6 ± 2.6	17.3 ± 1.8	17.6 ± 1.8	0.964
Backfat thickness, mm	7.8 ± 1.7	7.4 ± 1.2	8.5 ± 1.4	0.421
GR value, mm	10.5 ± 1.4	10.5 ± 1.3	11.1 ± 1.4	0.713

Note: Animal-level *p* values and, where applicable, superscript letters are exploratory. All variables were analyzed using one-way ANOVA, followed by Duncan’s multiple range test where appropriate. Different lowercase superscript letters within a row indicate nominal pairwise differences (*p* < 0.05).

**Table 5:** Organ weights and indices of Kazakh lambs across dietary groups.

Item	Organ	WS	CSH	FCSH	*p* Value
Organ weight, g	Heart	155.7 ± 40.0	165.8 ± 19.6	181.8 ± 27.4	0.345
	Lung	352.7 ± 58.1	356.5 ± 62.4	341.0 ± 37.2	0.874
	Spleen	50.5 ± 8.9	45.3 ± 4.5	60.6 ± 13.8	0.055
	Liver	567.8 ± 40.7	594.5 ± 85.4	586.3 ± 67.0	0.782
	Kidney	109.8 ± 6.8	127.2 ± 26.1	120.5 ± 8.1	0.071
Organ index, g/kg	Heart	4.1 ± 1.1	4.1 ± 0.4	4.3 ± 0.8	0.833
	Lung	9.2 ± 1.4	8.8 ± 1.6	8.1 ± 0.9	0.392
	Spleen	1.3 ± 0.2	1.1 ± 0.1	1.5 ± 0.4	0.116
	Liver	14.8 ± 0.9	14.7 ± 1.8	14.0 ± 1.9	0.625
	Kidney	2.9 ± 0.2	3.1 ± 0.6	2.9 ± 0.2	0.778

Note: Animal-level *p* values are exploratory. Kidney weight and kidney index were analyzed using the Kruskal–Wallis test, and all other variables using one-way ANOVA.

**Table 6:** Meat-quality traits of Kazakh lambs across dietary groups.

Item			WS	CSH	FCSH	*p* Value
Meat color	45 min	L*	31.5 ± 1.0	31.8 ± 1.9	30.6 ± 1.3	0.326
		a*	20.4 ± 1.6	20.2 ± 1.1	20.6 ± 1.7	0.914
		b*	6.0 ± 0.5	5.7 ± 0.3	6.0 ± 0.5	0.426
	24 h	L*	41.4 ± 1.6	40.4 ± 1.6	41.1 ± 2.0	0.646
		a*	18.2 ± 2.5	18.1 ± 1.6	19.4 ± 2.6	0.539
		b*	9.5 ± 2.0	7.6 ± 2.4	8.6 ± 1.1	0.258
pH		45 min	6.5 ± 0.4	6.7 ± 0.3	6.6 ± 0.3	0.710
		24 h	5.8 ± 0.1	5.7 ± 0.2	5.7 ± 0.1	0.503
Cooking loss, %			37.6 ± 2.5	37.0 ± 3.4	37.5 ± 1.9	0.917
Drip loss, %			1.7 ± 0.2	1.9 ± 0.6	1.9 ± 0.3	0.691
Shear force, N			52.6 ± 13.9	52.5 ± 13.1	62.9 ± 14.7	0.355

Note: Animal-level *p* values are exploratory. The 24-h pH was analyzed using the Kruskal–Wallis test. All other variables were analyzed using one-way ANOVA.

**Table 7:** Proximate composition of the longissimus thoracis of Kazakh lambs across dietary groups.

Item	WS	CSH	FCSH	*p* Value
Moisture, %	74.5 ± 0.2 ^a^	73.9 ± 1.3 ^ab^	73.1 ± 0.9 ^b^	0.021
Protein, %	21.2 ± 0.4	21.0 ± 0.5	21.1 ± 1.2	0.930
Fat, %	1.9 ± 0.1 ^b^	2.1 ± 0.2 ^ab^	2.2 ± 0.1 ^a^	0.013

Note: Animal-level *p* values and, where applicable, superscript letters are exploratory. Moisture was analyzed using Welch ANOVA followed by Games–Howell post hoc comparisons. Protein and fat were analyzed using one-way ANOVA; Duncan’s test was applied when the omnibus test was significant.

**Table 8:** Hydrolyzed amino acid composition of the longissimus thoracis of Kazakh lambs across dietary groups.

Item	WS	CSH	FCSH	*p* Value	*q* Value
Essential amino acids					
Threonine (Thr), mg/g	9.65 ± 0.46	9.65 ± 0.28	9.49 ± 0.62	0.795	0.845
Valine (Val), mg/g	9.58 ± 0.35 ^a^	9.31 ± 0.21 ^ab^	8.93 ± 0.35 ^b^	0.009	0.079
Methionine (Met), mg/g	5.26 ± 0.32	5.23 ± 0.16	5.01 ± 0.17	0.151	0.354
Isoleucine (Ile), mg/g	8.95 ± 0.37 ^a^	8.80 ± 0.17 ^ab^	8.36 ± 0.42 ^b^	0.024	0.139
Leucine (Leu), mg/g	16.08 ± 0.65	15.98 ± 0.46	15.50 ± 0.72	0.253	0.430
Phenylalanine (Phe), mg/g	7.39 ± 0.34 ^a^	7.42 ± 0.20 ^a^	7.00 ± 0.29 ^b^	0.038	0.159
Histidine (His), mg/g	9.90 ± 1.03	8.70 ± 0.73	9.48 ± 1.17	0.139	0.354
Lysine (Lys), mg/g	17.67 ± 0.68	17.46 ± 0.53	16.88 ± 0.85	0.165	0.354
Nonessential and conditionally essential amino acids					
Aspartic acid (Asp), mg/g	19.96 ± 1.12	20.27 ± 0.62	19.79 ± 0.90	0.661	0.750
Serine (Ser), mg/g	7.97 ± 0.33	7.92 ± 0.29	7.84 ± 0.59	0.861	0.861
Glutamic acid (Glu), mg/g	32.53 ± 1.62	32.53 ± 0.87	31.35 ± 2.13	0.372	0.527
Glycine (Gly), mg/g	8.47 ± 0.27	8.43 ± 0.31	8.17 ± 0.26	0.167	0.354
Alanine (Ala), mg/g	11.39 ± 0.41	11.36 ± 0.33	10.99 ± 0.52	0.231	0.430
Cysteine (Cys), mg/g	0.05 ± 0.02	0.03 ± 0.01	0.04 ± 0.02	0.587	0.713
Tyrosine (Tyr), mg/g	6.56 ± 0.34	6.58 ± 0.21	6.41 ± 0.29	0.550	0.713
Arginine (Arg), mg/g	11.85 ± 1.01	12.15 ± 0.43	11.47 ± 0.54	0.279	0.431
Proline (Pro), mg/g	8.10 ± 0.50 ^a^	7.51 ± 0.38 ^b^	7.28 ± 0.12 ^b^	0.005	0.079
Total amino acids (TAA), mg/g	191.34 ± 7.42	189.35 ± 4.90	184.00 ± 8.74	0.222	n/a
Essential amino acids (EAA), mg/g	84.47 ± 3.06	82.56 ± 1.82	80.65 ± 4.03	0.138	n/a
Non-essential and conditionally essential amino acids (NEAA + CEAA), mg/g	106.87 ± 4.91	106.80 ± 3.19	103.35 ± 4.89	0.314	n/a
EAA/TAA, %	44.15 ± 0.80	43.60 ± 0.34	43.83 ± 0.47	0.278	n/a

Note: Animal-level *p* values and, where applicable, superscript letters are exploratory. Cysteine was analyzed using the Kruskal–Wallis test, and all other variables using one-way ANOVA, followed by Duncan’s multiple range test where appropriate. Different lowercase superscript letters within a row indicate nominal unadjusted pairwise differences (*p* < 0.05). The *q* values were calculated using the Benjamini–Hochberg FDR correction across the 17 individual amino acids; none reached *q* < 0.05. The four summary indices were excluded from FDR correction (n/a, not applicable). TAA is the sum of the 17 detected amino acids. EAA includes Thr, Val, Met, Ile, Leu, Phe, His, and Lys; Trp was not measured in this analysis.

## Data Availability

The original contributions presented in this study are included in the article/Supplementary Material. Further inquiries can be directed to the corresponding author.

## Supplementary Materials

The following supporting information can be downloaded at: https://www.mdpi.com/article/10.3390/foods15203739/s1. Table S1. GC–IMS volatile-signal peak volumes and exploratory statistical results across the three dietary treatments; Table S2. GC–IMS M/D-combined peak volumes and exploratory statistical results across the three dietary treatments. Table S3. Pearson Correlations between Hydrolyzed Amino Acids and Candidate GC–IMS Volatile Signals with BH-FDR Correction. Table S4. Distributional diagnostics and statistical methods used for outcomes reported in Table 2, Table 3, Table 4, Table 5, Table 6, Table 7 and Table 8. Supplementary Methods S1. Plasma Antioxidant Assays; Data S1. GC–IMS Peak-Volume Data Used for Statistical Analysis.

## Abbreviations

The following abbreviations are used in this manuscript:

WS	wheat straw
CSH	cottonseed hulls
FCSH	fermented cottonseed hulls
ADF	acid detergent fiber
ALB	albumin
ALT	alanine aminotransferase
ANOVA	analysis of variance
AST	aspartate aminotransferase
BH-FDR	Benjamini–Hochberg false discovery rate
CAS	Chemical Abstracts Service
CAT	catalase
CEAA	conditionally essential amino acids
EAA	essential amino acids
FDR	false discovery rate
GC-IMS	gas chromatography–ion mobility spectrometry
GGT	gamma-glutamyl transferase
GSH	glutathione
GSH-Px	glutathione peroxidase
GPx	glutathione peroxidase
GPX1	glutathione peroxidase 1
GPX4	glutathione peroxidase 4
LSD	least significant difference
M	monomer
D	dimer
MDA	malondialdehyde
NDF	neutral detergent fiber
NEAA	nonessential amino acids
NIST	National Institute of Standards and Technology
NH_3_-N	ammonia nitrogen
PCA	principal component analysis
PC1	first principal component
PC2	second principal component
RI	retention index
SOD	superoxide dismutase
TAA	total amino acids
T-AOC	total antioxidant capacity
TNF-α	tumor necrosis factor alpha
T-SOD	total superoxide dismutase
