# Supplementary material for: Carcass Traits, Meat Quality, Hydrolyzed Amino Acid Composition, and GC–IMS Volatile Signal Profiles in Kazakh Lambs Fed Diets Containing Fermented Cottonseed Hulls
Source: Foods. 2026 Oct 20;15(20):3739. doi: 10.3390/foods15203739 (PMC13704721; doi:10.3390/foods15203739)
Supplement: Supplementary file 1 [file foods-15-03739-s001.zip › Supplementary Methods S1.Plasma Antioxidant Assays.pdf]

Supplementary Methods S1. Plasma Antioxidant Assays

Plasma samples from 30 Kazakh lambs were analyzed using commercial colorimetric assay kits from Nanjing Jiancheng Bioengineering Institute (Nanjing, China). Each biological sample was assayed in three parallel technical reactions, and the mean value was used for statistical analysis. Assay-specific controls, blanks, and calibration standards were included as described below. The procedures reflect the analytical records for this study.

S1.1. Total superoxide dismutase (T-SOD)

T-SOD activity was determined using a xanthine oxidase–hydroxylamine colorimetric assay (kit A001-1). Plasma (20 μL) was added to the assay tube; 20 μL of distilled water was used in the corresponding control tube. Each tube received 1.0 mL of reagent 1 and 0.1 mL each of reagents 2, 3, and 4. After thorough mixing, the tubes were incubated at 37 °C for 40 min. Chromogenic reagent (2.0 mL) was added, followed by incubation at room temperature for 10 min. Absorbance was measured at 550 nm against distilled water using a 1-cm optical path. Activity was calculated from inhibition relative to the control, with one unit (U) defined in the laboratory protocol as the amount of SOD giving 50% inhibition, and expressed as U/mL plasma.

S1.2. Glutathione peroxidase (GSH-Px)

GSH-Px activity was measured using a glutathione-consumption colorimetric assay (kit A005-1). The enzyme and non-enzyme reaction tubes each received 0.2 mL of 1 mmol/L reduced glutathione (GSH). Plasma (0.1 mL) was added to the enzyme tube, and the tubes were prewarmed at 37 °C for 5 min. Reagent 1 (0.1 mL, prewarmed) was added, and the reactions proceeded at 37 °C for 5 min. Reagent 2 (2.0 mL) was added to stop the reaction, and 0.1 mL of plasma was then added to the non-enzyme tube. After mixing, samples were centrifuged at 4000 rpm for 10 min. For color development, 1.0 mL supernatant or GSH reference solution was mixed with 1.0 mL reagent 3, 0.25 mL reagent 4, and 0.05 mL reagent 5. Absorbance was recorded at 412 nm after 15 min, using a 20 μmol/L GSH reference and a blank. Activity was calculated after subtracting the non-enzymatic GSH consumption and expressed as U/mL plasma; the laboratory protocol defines one unit as a decrease of 1 μmol/L GSH per minute at 37 °C per mL plasma.

S1.3. Catalase (CAT)

CAT activity was determined using an ammonium molybdate colorimetric assay (kit A007-1-1). For the sample reaction, 0.1 mL plasma was mixed with 1.0 mL prewarmed reagent 1 and 0.1 mL prewarmed reagent 2 and incubated at 37 °C for 6 min. Reagent 3 (1.0 mL) and reagent 4 (0.1 mL) were then added. For the corresponding control, 0.1 mL plasma was added after the reaction step. After mixing, 200 μL of each reaction mixture was transferred to a 96-well plate and absorbance was measured at 405 nm. Activity was calculated from the difference between control and sample absorbance using the recorded reaction time (360 s) and laboratory assay coefficient and expressed as U/mL plasma.

S1.4. Malondialdehyde (MDA)

MDA concentration was measured by the thiobarbituric acid (TBA) colorimetric method (kit A003-1). Sample, standard, and blank tubes received 0.05 mL plasma, 0.05 mL of a 10 nmol/mL MDA standard, or 0.05 mL absolute ethanol, respectively. Each tube was mixed with 0.05 mL reagent 1, 0.75 mL reagent 2, and 0.25 mL reagent 3. After mixing, the tubes were heated at 95 °C for 40 min, cooled under running water, and centrifuged at 3500 rpm for 10 min. Supernatant absorbance was measured at 532 nm. MDA content was calculated from the sample, blank, and

standard absorbance values and reported as nmol/mL plasma.

S1.5. Total antioxidant capacity (T-AOC)

T-AOC was determined using the ABTS colorimetric microplate method (kit A015-2-1). Plasma was diluted fourfold, and 10 μL of diluted sample was mixed with 20 μL of reagent 4 working solution and 170 μL of ABTS working solution. Blank wells contained 10 μL distilled water instead of sample, while standard wells contained 10 μL Trolox solution. After incubation for 6 min at room temperature, absorbance was read at 405 nm. A Trolox calibration series of 0, 0.1, 0.2, 0.4, 0.8, and 1.0 mmol/L was used. T-AOC was calculated from the calibration curve with correction for the fourfold dilution and expressed as mmol Trolox equivalents/L plasma.
